# Synthesis, crystal structure and Hirshfeld surface analysis of (2*Z*,2′*E*)-2,2′-(3-meth­oxy-3-phenylpropane-1,2-diyl­idene)bis­(hydrazine-1-carbo­thioamide) di­methyl­formamide monosolvate

**DOI:** 10.1107/S2056989023007946

**Published:** 2023-09-14

**Authors:** Firudin I. Guseinov, Aleksandr V. Knyazev, Elena V. Shuvalova, Konstantin I. Kobrakov, Aida I. Samigullina, Zeliha Atioğlu, Mehmet Akkurt, Ajaya Bhattarai

**Affiliations:** a Kosygin State University of Russia, 117997 Moscow, Russian Federation; b N. D. Zelinsky Institute of Organic Chemistry, Russian Academy of Sciences, 119991 Moscow, Russian Federation; c MIREA, Russian Technology University, Lomonosov Institute of Fine Chemical Technology, Moscow, 119571, Russian Federation; d Department of Aircraft Electrics and Electronics, School of Applied Sciences, Cappadocia University, Mustafapaşa, 50420 Ürgüp, Nevşehir, Türkiye; e Department of Physics, Faculty of Sciences, Erciyes University, 38039 Kayseri, Türkiye; f Department of Chemistry, M.M.A.M.C (Tribhuvan University), Biratnagar, Nepal; Universität Greifswald, Germany

**Keywords:** crystal structure, disorder, hydrogen bonds, Hirshfeld surface analysis, α-chloro­ketone, chloro­oxirane, bis­thio­semi­carbazone

## Abstract

The overall mol­ecular configuration of the title compound, C_12_H_16_N_6_OS_2_·C_3_H_7_NO, is stabilized in the solid state by intra­molecular C—H⋯N, C—H⋯O, N—H⋯N and N—H⋯O inter­actions, forming *S*(5) ring motifs. In the crystal, mol­ecules are linked to each other and solvent di­methyl­formamide mol­ecules by N—H⋯S, N—H⋯O, C—H⋯O and C—H⋯S hydrogen bonds, forming a three dimensional network. The phenyl ring of the title compound is disordered over two sites with an occupancy ratio of 0.57 (4):0.43 (4). A Hirshfeld surface analysis was performed to qu­antify the contributions of the different inter­molecular inter­actions, indicating that the most important contributions to the crystal packing are from H⋯H (38.7%), S⋯H / H⋯S (24.0%), C⋯H / H⋯C (18.5%) and N⋯H / H⋯N (9.8%) inter­actions.

## Abstract 2

In the crystal of the title compound, mol­ecules are linked to each other and solvent di­methyl­formamide mol­ecules by N—H⋯S, N—H⋯O, C—H⋯O and C—H⋯S hydrogen bonds, forming a three dimensional network.

## 1. Chemical context

Hydrazones are very attractive compounds in synthesis, catalysis, crystal engineering and medicinal chemistry due to their reactivity, hydrogen-bonding donor ability and broad spectrum of biological activities (Afkhami *et al.*, 2019 ▸; Gurbanov *et al.*, 2020*a* ▸,*b* ▸; Mahmoudi *et al.*, 2017*a* ▸,*b* ▸,*c* ▸; Khalilov 2021 ▸; Martins *et al.*, 2017 ▸). The most common synthetic pathway for the synthesis of hydrazones is the reaction of appropriate hydrazines with different aldehydes or ketones in various organic solvents (Khalilov *et al.*, 2021 ▸). For example, hydrazinecarbo­thio­amide has been well explored as a substrate in the synthesis of hydrazones (Safarova *et al.*, 2019 ▸; Velásquez *et al.*, 2019 ▸). The functional properties of hydrazones can be improved by attaching electron-withdrawing or -donating substituents to the hydrazone moiety (Gurbanov *et al.*, 2022*b* ▸, 2017 ▸, 2021 ▸; Kopylovich *et al.*, 2011 ▸). In fact, due to the participation of the substituents in various sorts of inter­molecular inter­actions (Mahmudov *et al.*, 2010 ▸, 2012 ▸, 2022 ▸; Mahmoudi *et al.*, 2019 ▸, 2021 ▸) the catalytic activity of metal complexes of hydrazones has been improved in comparison to those with unsubstituted ligands (Gurbanov *et al.*, 2022*a* ▸). In order to continue our work in this perspective, we have synthesized a new hydrazone di­methyl­formamide monosolvate, (2*Z*,2′*E*)-2,2′-(3-meth­oxy-3-phenyl­propane-1,2-diyl­idene)bis­(hydrazine-1-carbo­thio­amide)·DMF *via* reaction of hydrazinecarbo­thio­amide with the highly reactive substrate 2-chloro-2-(di­eth­oxy­meth­yl)-3-phenyl­oxirane, which may be also replaced by 1-chloro-3,3-dieth­oxy-1-phenyl­propan-2-one (Guseinov *et al.*, 2006 ▸, 2017 ▸, 2020 ▸). 
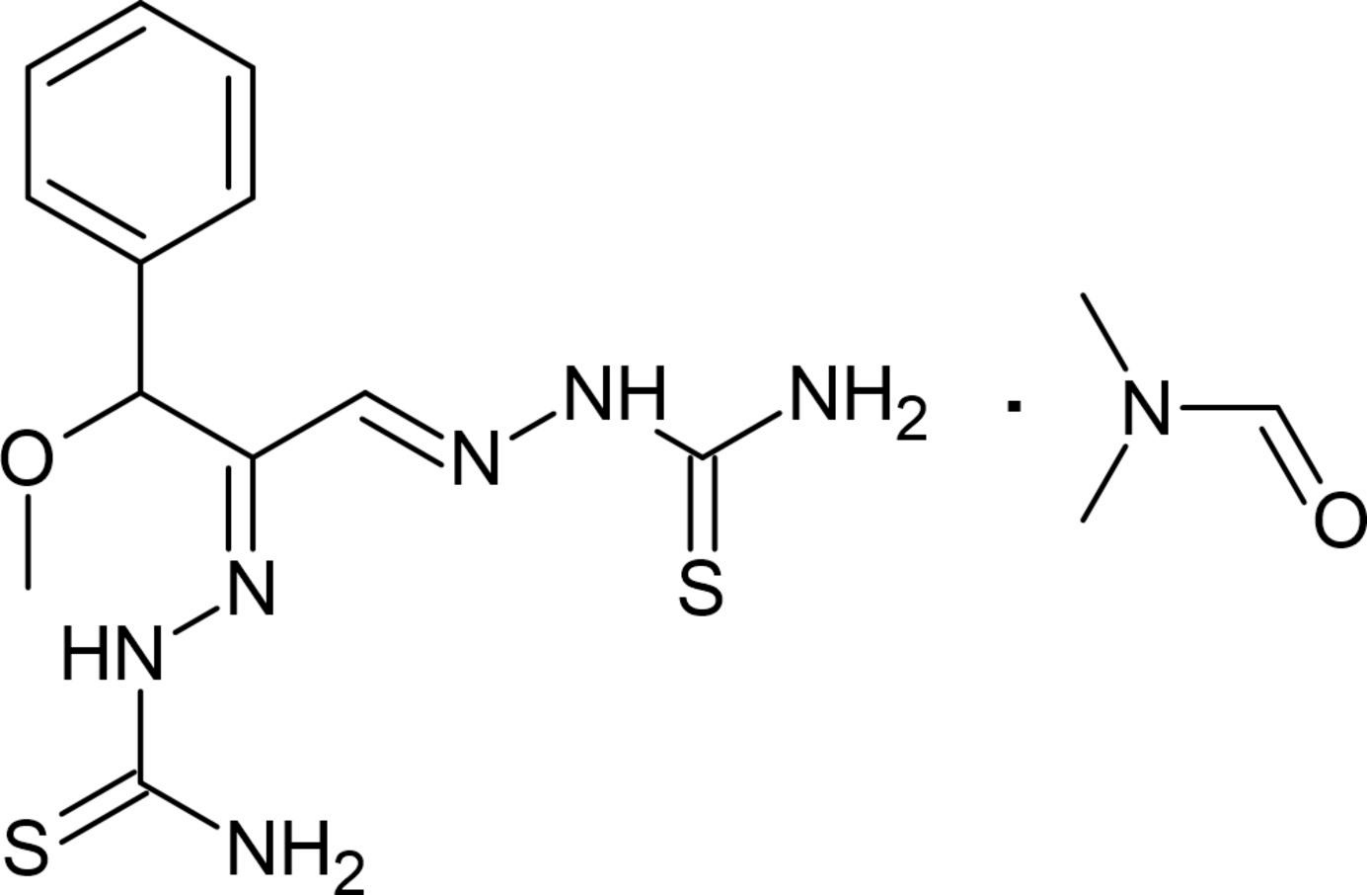


## 2. Structural commentary

**Figure 1:**
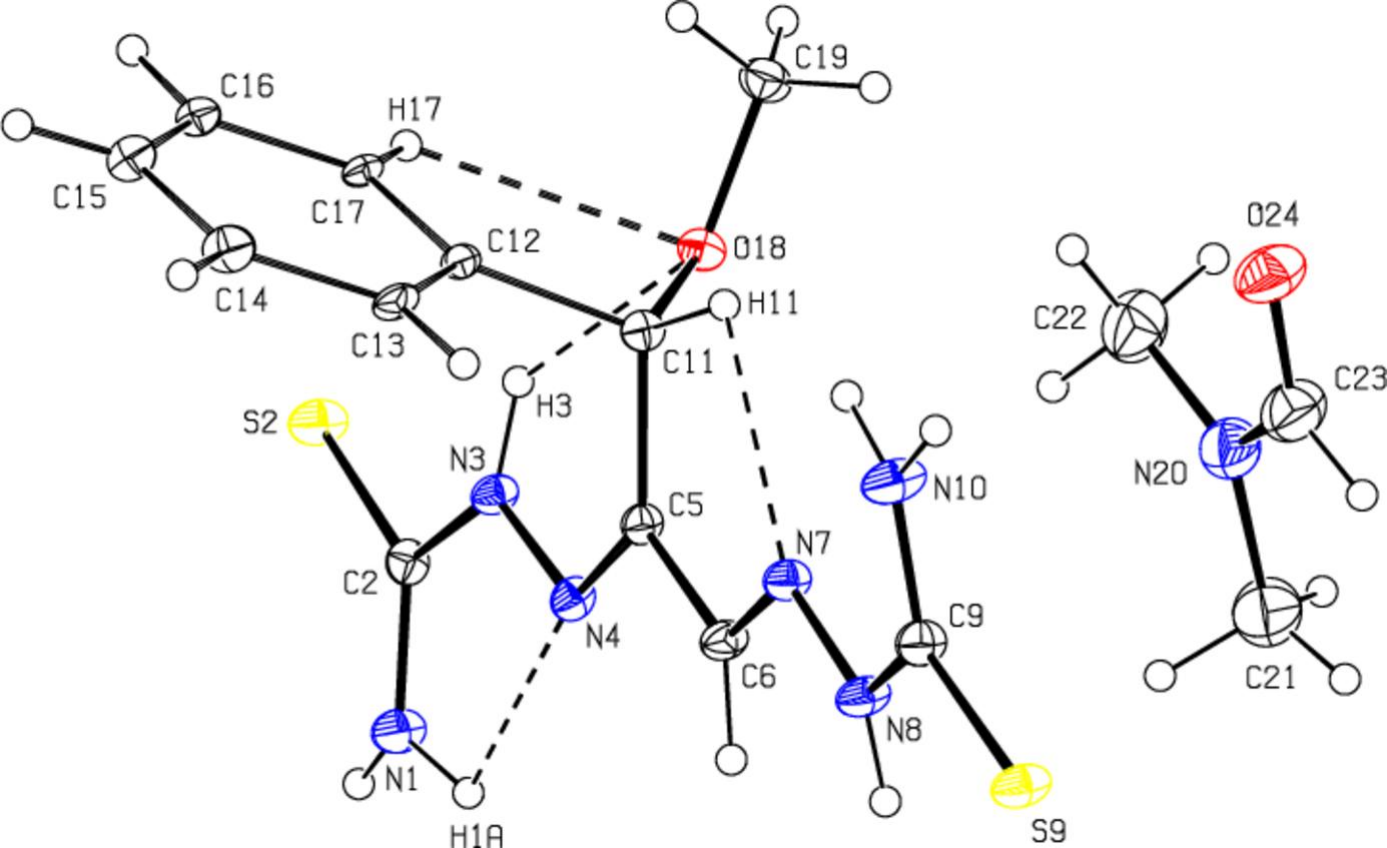
The mol­ecular structure of the title compound, showing the atom labeling and displacement ellipsoids drawn at the 30% probability level. Intra­molecular C11—H11⋯N7, C17—H17⋯O18, N1—H1*A*⋯N4 and N3—H3⋯O18 inter­actions are shown as dashed lines. The minor component of the disorder was omitted for clarity reasons.

As shown in Fig. 1 ▸, the title compound adopts a *Z* configuration about the C5=C6 double bond with regard to the 3-meth­oxy-3-phenyl­propane group and *E* configuration regarding the hydrazine-1-carbo­thio­amide moieties. The bond has a length of 1.452 (3) Å. The mol­ecular conformation of the title compound is stabilized by intra­molecular C11—H11⋯N7, C17—H17⋯O18, N1—H1*A*⋯N4 and N3—H3⋯O18 classical and non-classical hydrogen-bonding inter­actions, resulting in *S*(5) ring motifs (Table 1 ▸; Bernstein *et al.*, 1995 ▸). The C12–C17 phenyl ring is disordered over two sites with occupancy factors in a 0.57 (4) to 0.43 (4) ratio. The major (C12–C17) and minor (C12*A*–C17*A*) components of the disordered phenyl ring subtend a dihedral angle of 2.0 (9)° to each other, *i.e*. they are nearly co-planar. Bond lengths and angles of the title compound are generally in agreement with those reported for related compounds, as discussed in the *Database survey* section below.

## 3. Supra­molecular features and Hirshfeld surface analysis

**Figure 2:**
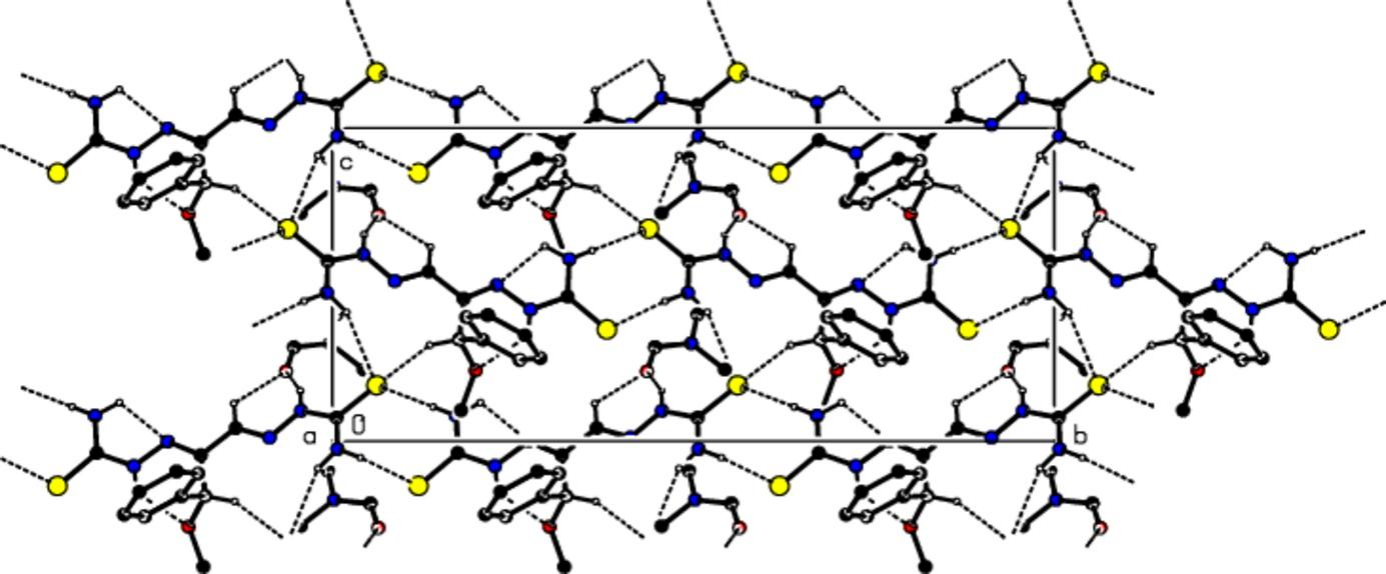
View of the mol­ecular packing along the *a*-axis. Intra­molecular C—H⋯N, C—H⋯O, N—H⋯N and N—H⋯O inter­actions and inter­molecular N—H⋯S, N—H⋯O, C—H⋯O and C—H⋯S hydrogen bonds are shown as dashed lines. The minor part of the disorder and hydrogen atoms not involved in hydrogen bonding were omitted for clarity reasons.

**Figure 5:**
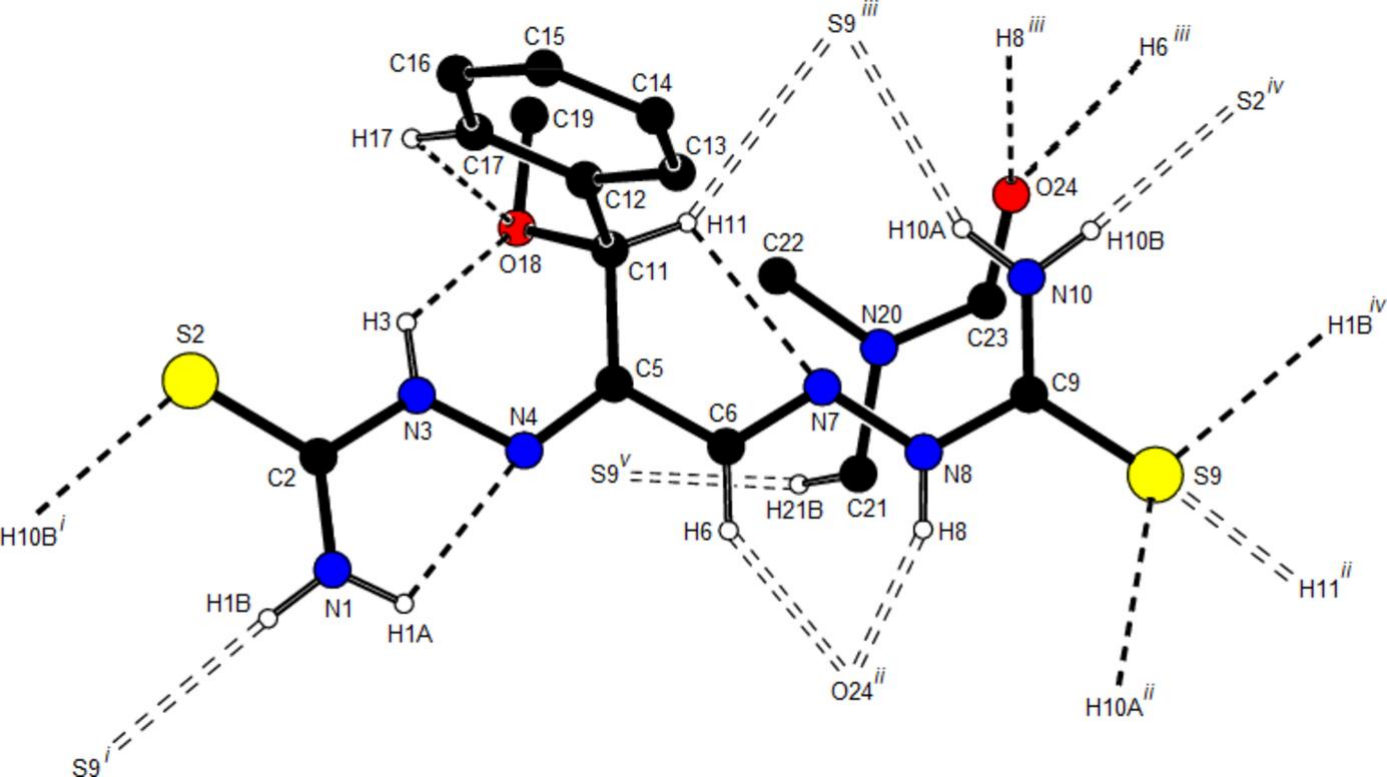
A general view of the possible intra- and inter­molecular hydrogen bonds of the mol­ecule. The minor disorder component was omitted for clarity. Symmetry codes: (i) *x* − 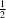 , *y* + 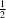 , *z*; (ii) *x*, −*y* + 1, *z* + 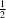 ; (iii) *x*, −*y* + 1, *z* − 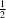 ; (iv) *x* + 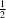 , *y* − 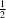 , *z*; (v) *x* − 1, −*y* + 1, *z* − 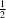 .

Mol­ecules in the crystal of the title compound are linked to each other and to the solvent di­methyl­formamide by classical and non-classical N—H⋯S, N—H⋯O, C—H⋯O and C—H⋯S hydrogen bonds (Table 1 ▸; Figs. 2 ▸, 3 ▸ and 4 ▸), resulting in a three-dimensional network. Fig. 5 ▸ shows all inter­actions as supplied in Table 1 ▸. In addition some offset weak C/N—H⋯π inter­actions are observed.

**Figure 6:**
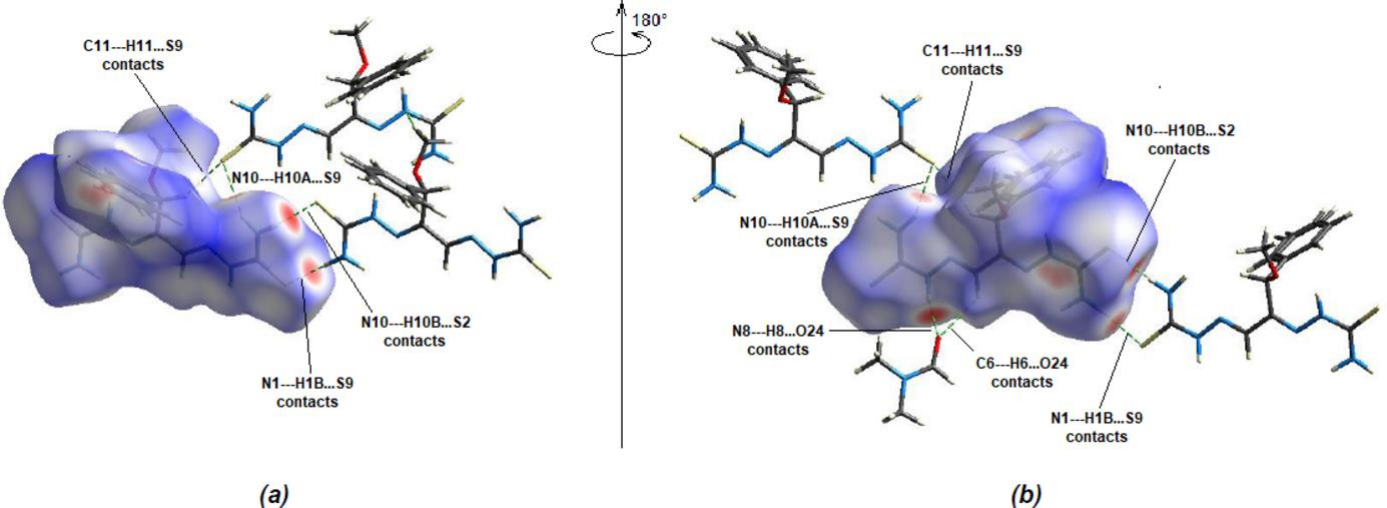
(*a*) Front and (*b*) back sides of the three-dimensional Hirshfeld surface of the title compound mapped over *d*_norm_.

*Crystal Explorer 17.5* (Spackman *et al.*, 2021 ▸) was used to perform a Hirshfeld surface analysis and to generate the corresponding two-dimensional fingerprint plots, with a standard resolution of the three-dimensional *d*_norm_ surfaces plotted over a fixed color scale of −0.5044 (red) to +1.5170 (blue) a.u. (Fig. 6 ▸). The red spots symbolize short contacts and negative *d*_norm_ values on the surface corresponding to the N—H⋯S, N—H⋯O and C—H⋯O hydrogen bonds mentioned above (Table 1 ▸). The N1—H1*B*⋯S9, N8—H8⋯O24, N10—H10*A*⋯S9, N10—H10*B*⋯S2 and C6—H6⋯O24 inter­actions, which play a key role in the mol­ecular packing of the title compound, are responsible for the red spots observed around S2, S9 and O24.

**Figure 7:**
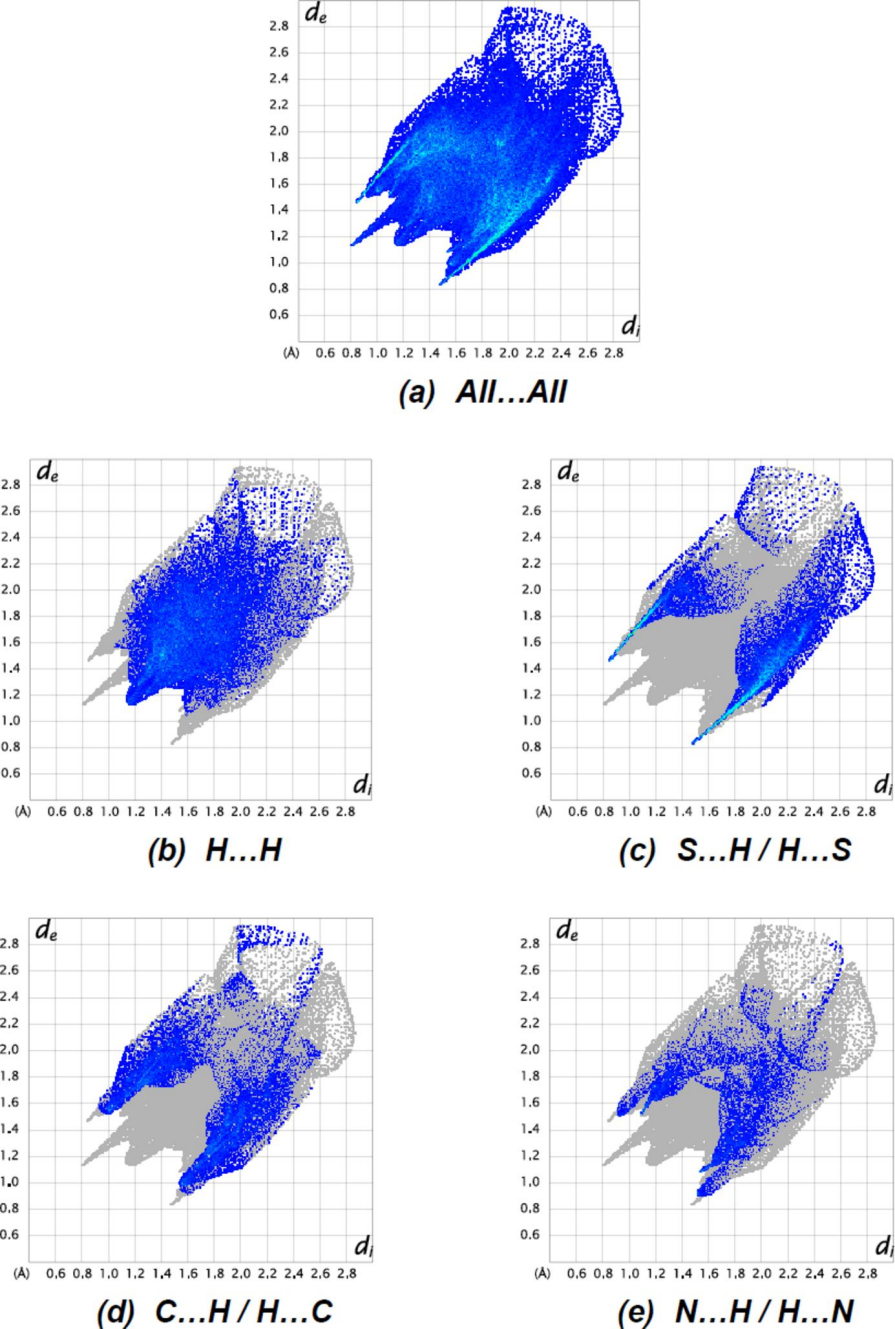
The two-dimensional fingerprint plots of the title compound, showing (*a*) all inter­actions, and those delineated into (*b*) H⋯H, (*c*) S⋯H/H⋯S, (*d*) C⋯H/H⋯C and (*e*) N⋯H/H⋯N inter­actions. [*d*_e_ and *d*_i_ represent the distances from a point on the Hirshfeld surface to the nearest atoms outside (external) and inside (inter­nal) the surface, respectively].

The overall two-dimensional fingerprint plot for the title compound is provided in Fig. 7 ▸ *a*, and those delineated into N—H⋯S, N—H⋯O and C—H⋯O contacts are shown in Fig. 7 ▸ *b*–*e*, while numerical details of the different contacts are supplied in Table 2 ▸. The most important contributions to the Hirshfeld surfaces from the various inter­atomic contacts are H⋯H (38.7%), S⋯H / H⋯S (24.0%), C⋯H/H⋯C (18.5%) and N⋯H/H⋯N ((9.8%). Other, less notable contacts comprise O⋯H/H⋯O (5.0%), S⋯N/N⋯S (1.7%), S⋯C/C⋯S (0.7%), O⋯N/N⋯O (0.5%), N⋯C/C⋯N (0.4%), N⋯N (0.2%), C⋯C (0.2%) and S⋯O/O⋯S (0.1%); they have little, if any, directional influence on the mol­ecular packing.

## 4. Database survey

A database search was carried out using *ConQUEST* (Bruno *et al.*, 2002 ▸), part of the software for version 2023.2.0 of the Cambridge Structural Database (Groom *et al.*, 2016 ▸). A search for the keyword ‘hydrazinecarbo­thio­amide’ resulted in nearly 600 hits. A search for the structural bis-hydrazinecarbo­thio­amide motif without considering hydrogen atoms narrowed that down to 45. For a more detailed analysis, four of those compounds were chosen as relatively more closely related to the title compound, yet with a variation of the substituent(s) numbers and position on the bis-hydrazinecarbo­thio­amide backbone. These are: (*E*,*E*)-*N*,*N*-dimethyl-2-{3-[(methyl­carbamo­thio­yl)hydrazono]butan-2-yl­idene}hydrazinecarbo­thio­amide (CD refcode ERABIJ; Paterson *et al.*, 2010 ▸), diacetyl-2-(4-*N*-ethyl-3-thio­semicarbazone)-3-(4-*N*-allyl-3-thio­semicarbazone) di­methyl­sulfoxide solvate (JEXXOA; Holland *et al.*, 2007 ▸), 2-keto-3-eth­oxy­butyraldehyde­bis­(thio­semicarbazone) (KEBASC10; Gabe *et al.*, 1969 ▸) and *N*,1-dimethyl-2-{3-[2-(methyl­carbamo­thio­yl)hydrazinyl­idene]butan-2-yl­idene}hydrazine-1-carbo­thio­amide (RECKAP; Alonso *et al.*, 2022 ▸).

In the crystal of ERABIJ (monoclinic space group: *P*2_1_/*c*, *Z* = 4), the mol­ecule adopts an (*E*, *E*)-configuration about the imine double bonds. The arm bearing a dimethyl substituent has a slightly shorter C—S [1.6802 (19) Å] bond length and a longer C—N [1.341 (2) Å] bond length than the arm with a single methyl substituent [1.693 (2) and 1.323 (3) Å, respectively]. These bond lengths indicate that there is some extensive delocalization throughout the mol­ecule while one tautomeric form still dominates.

In the crystal of JEXXOA (monoclinic space group: *P*2_1_/*c*, *Z* = 2), the unsymmetrical bis­(thio­semicarbazone) lies on a crystallographic center of inversion. The carbon–carbon bond length between C5 and C6 is 1.478 (3) Å, which is exactly the same as the average bond length expected for a single bond between two *sp* ^2^-hybridized carbon atoms. Other bond lengths are indicative of the presence of a conjugated system here as well.

In the crystal of KEBASC10 (monoclinic space group: *P*2_1_/*c*, *Z* = 8), there are two mol­ecules per asymmetric unit. The bis-hydrazinecarbo­thio­amide motif is outstretched (*i.e.* not bent) and extends from one sulfur atom to the other as head and tail atoms. The mol­ecule is approximately planar except for the side chain. The bond distances and angles are very similar in the two mol­ecules of the asymmetric unit. There is an intra­molecular N—H⋯O hydrogen bond, which stabilizes the mol­ecular structure, similar to what is observed in the title compound. The packing of the mol­ecule seems dominated by the formation of N—H⋯S hydrogen bonds. There is also one very short C—H⋯S inter­molecular distance between the two mol­ecules in the asymmetric unit, which may be strong enough to cause some distortion, in one mol­ecule more than in the other. The tendency of mol­ecules that are crystallographically independent but have opposite absolute configurations to associate may explain why they have co-crystallized in this case and why there are, hence, two independent mol­ecules in the asymmetric unit.

In the crystal of RECKAP (triclinic space group: *P* 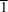 , *Z* = 2), the compound is in the thione form yet resonant, which is supported by the C—S bond distances, which are inter­mediate between those of single and double bonds (1.82 and 1.56 Å, respectively) and the presence of the hydrazinic hydrogen H2. The azomethine bonds both have a length of 1.29 Å, which is in accordance with double bonds. The N—N bonds are both shorter than 1.44 Å, which agrees well with those of similar thio­semicarbazones. The two arms of the mol­ecule adopt the *E* configuration with respect to the central C3—C4 single bond and both azomethine nitro­gen atoms N3 and N4 are in an *E* configuration relative to the thione sulfur atoms. The ligand is not planar and the two arms form an angle of 73.51°. The mol­ecules are held together in the crystal through an extended network of inter­molecular hydrogen bonds involving the amine nitro­gen atoms N1 and N6 and the sulfur atoms.

All the mol­ecules discussed here, including the title compound, adopt an *E* configuration of the hydrazine moieties attached to the central C—C bond. The differences in substitution do not affect this. However, the latter gives rise to a variation in the inter­molecular inter­actions and can also result in distinct mol­ecular shapes from the more common (almost) planar arrangement of the bis-hydrazinecarbo­thio­amide motif to a substantial twisting to nearly perpendicular.

## 5. Synthesis and crystallization

Hydrazinecarbo­thio­amide (0.380 g, 6.25 mmol) and 2-chloro-2-(di­eth­oxy­meth­yl)-3-phenyl­oxirane (1.600 g, 6.25 mmol) in 20 mL of methanol was refluxed for 2 h. After complete dissolution of hydrazinecarbo­thio­amide, the mixture was stirred at room temperature for 24 h. The progress of the reaction was monitored by TLC in the system 9:1 chloro­form:methanol *R*_f_ = 0.53. After completion of the reaction, the solvent was evaporated. The title compound was isolated by column chromatography in a 20:1 chloro­form:methanol *R*_f_ = 0.17 system. The compound was obtained as a white solid in a yield of 0.689 g (34%); m.p. 421–423 K (with decomposition). Analysis calculated for C_12_H_16_N_6_OS_2_ (*M* = 324.42) C 44.43, H 4.97, N 25.91; found: C 44.35, H 4.90, N 25.94. ^1^H NMR (300 MHz, DMSO-*d*_6_) *δ* 3.52 (3H, CH_3_), 6.35 (1H, CH), 7.37–7.45 (5H, Ar), 8,15–8.68 (2H, NH), 10.74 (2H, NH_2_), 10.80 (*s*, 2H, NH_2_). ^13^C NMR (200 MHz, DMSO-*d*_6_) δ 57.13, 78.52, 126.22, 128.45, 136.79, 138.92, 144.20, 177.67, 178.18. Crystals suitable for X-ray diffraction analysis were obtained by slow evaporation of the DMF:methanol solution.

## 6. Refinement

Crystal data, data collection and structure refinement details are summarized in Table 3 ▸. The C12(C12*A*)–C17(C17*A*) atoms in the C12–C17 phenyl ring are disordered over two sites with occupancies of 0.57 (4) and 0.43 (4), respectively. The N-bound hydrogen atoms were located in difference maps [N1—H1*A* = 0.92 (2), N1—H1*B* = 0.92 (2), N3—H3 = 0.90 (2), N8—H8 = 0.88 (2), N10—H10*A* = 0.91 (2) and N10—H10*B* = 0.90 (2) Å] and refined by constraining the N—H distances with SADI. All carbon-bound hydrogen atoms were positioned geometrically (C—H = 0.95–1.00 Å) and were included in the refinement in the riding-model approximation with *U*_iso_(H) = 1.2 or 1.5*U*_eq_(C).

## Supplementary Material

Crystal structure: contains datablock(s) I. DOI: 10.1107/S2056989023007946/yz2041sup1.cif

Structure factors: contains datablock(s) I. DOI: 10.1107/S2056989023007946/yz2041Isup2.hkl

CCDC reference: 2294475

Additional supporting information:  crystallographic information; 3D view; checkCIF report

## Figures and Tables

**Figure 3:**
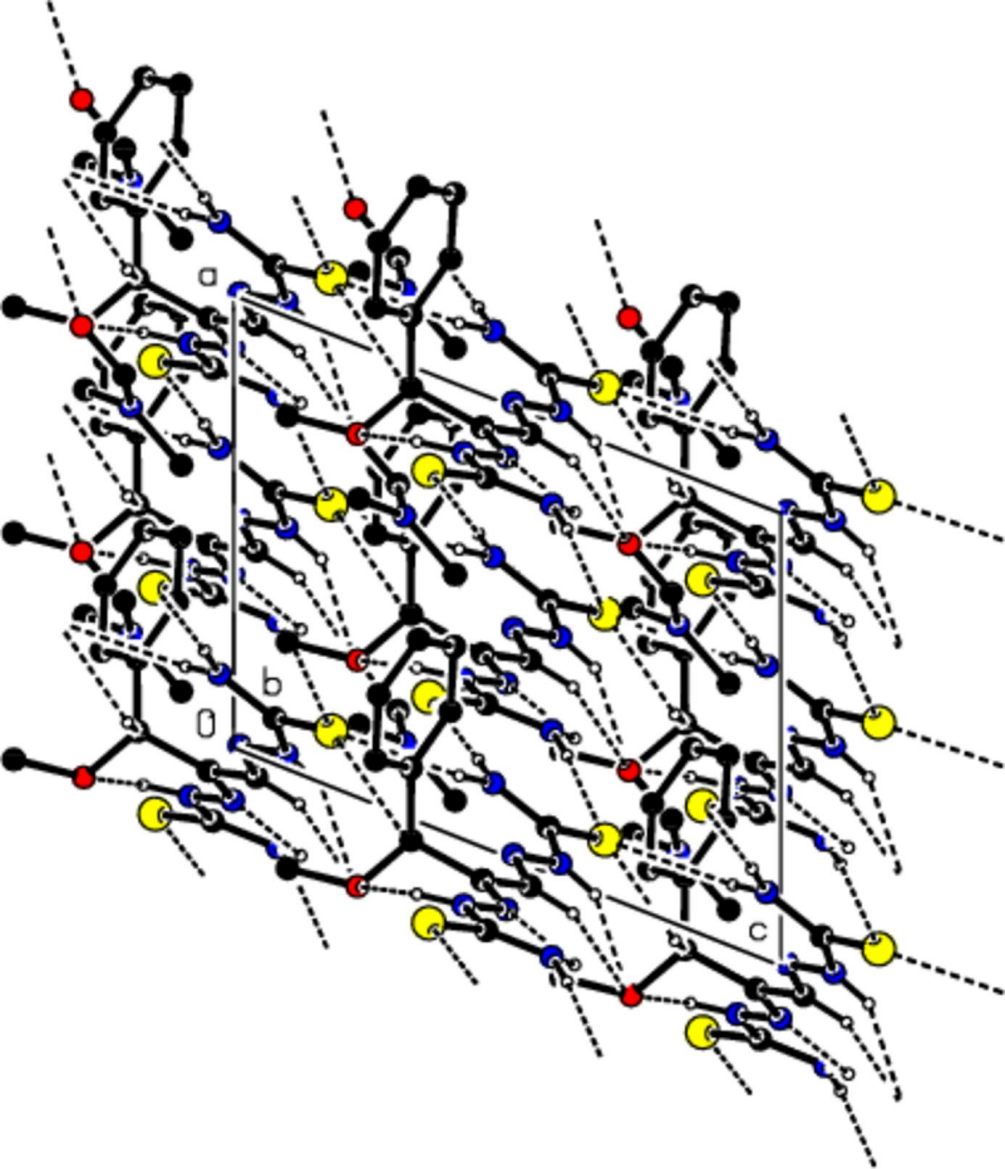
View of the mol­ecular packing along the *b*-axis. Intra­molecular C—H⋯N, C—H⋯O, N—H⋯N and N—H⋯O inter­actions and inter­molecular N—H⋯S, N—H⋯O, C—H⋯O and C—H⋯S hydrogen bonds are shown as dashed lines. The minor part of the disorder and hydrogen atoms not involved in hydrogen bonding were omitted for clarity reasons.

**Figure 4:**
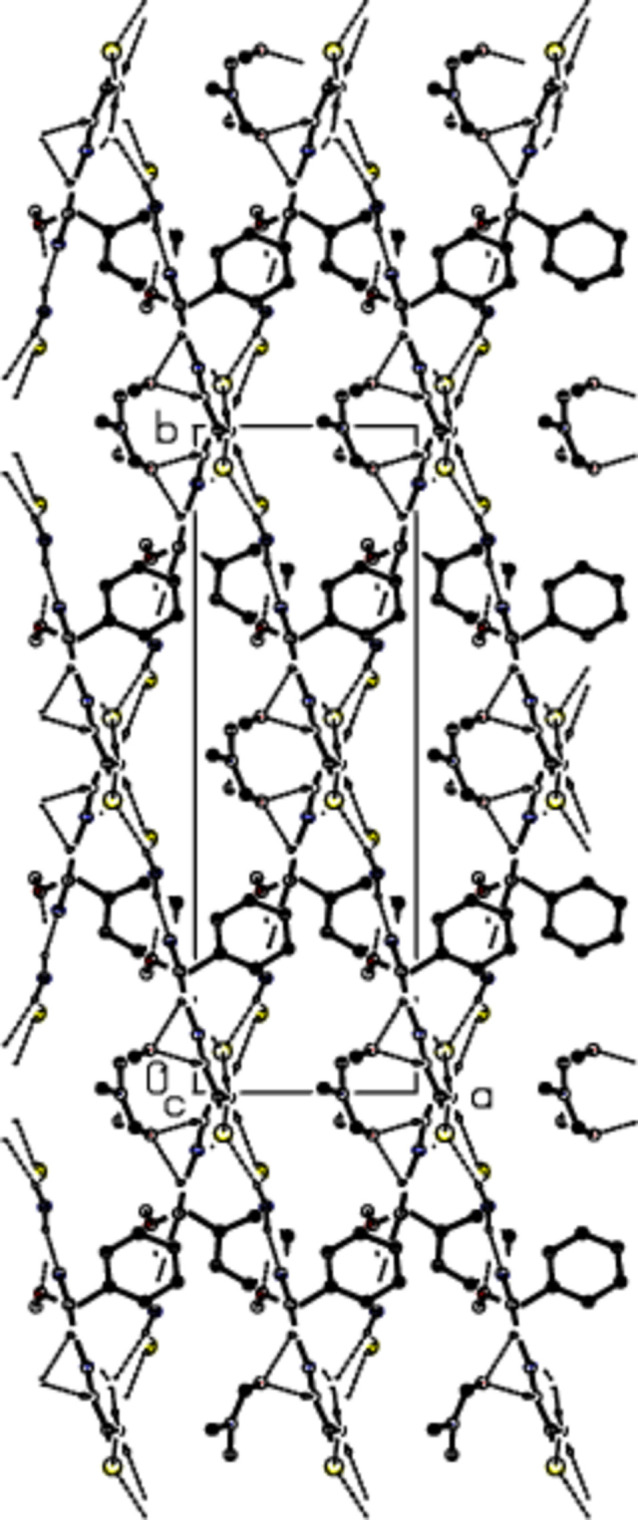
View of the mol­ecular packing along the *c*-axis. Intra­molecular C—H⋯N, C—H⋯O, N—H⋯N and N—H⋯O inter­actions and inter­molecular N—H⋯S, N—H⋯O, C—H⋯O and C—H⋯S hydrogen bonds are shown as dashed lines. The minor part of the disorder and hydrogen atoms not involved in hydrogen bonding were omitted for clarity reasons.

**Table 1:** Hydrogen-bond geometry (Å, °)

*D*—H⋯*A*	*D*—H	H⋯*A*	*D*⋯*A*	*D*—H⋯*A*
N1—H1*A*⋯N4	0.92 (3)	2.27 (4)	2.670 (3)	106 (3)
N1—H1*B*⋯S9^i^	0.92 (4)	2.54 (4)	3.450 (2)	172 (4)
N3—H3⋯O18	0.90 (2)	2.04 (3)	2.694 (3)	129 (3)
N8—H8⋯O24^ii^	0.87 (3)	2.07 (4)	2.847 (4)	149 (3)
N10—H10*A*⋯S9^iii^	0.91 (3)	2.72 (3)	3.570 (3)	156 (3)
N10—H10*B*⋯S2^iv^	0.91 (4)	2.42 (4)	3.322 (3)	175 (4)
C6—H6⋯O24^ii^	0.95	2.54	3.224 (4)	129
C11—H11⋯N7	1.00	2.42	2.853 (3)	105
C11—H11⋯S9^iii^	1.00	2.87	3.706 (2)	141
C17—H17⋯O18	0.95	2.45	2.787 (16)	101
C21—H21*B*⋯S9^v^	0.98	2.98	3.951 (5)	171

Symmetry codes: (i) 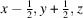 ; (ii) 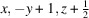 ; (iii) 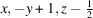 ; (iv) 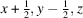 ; (v) 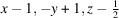 .

**Table 2:** Summary of short inter­atomic contacts (Å) in the title compound

Contact	Distance	Symmetry operation
S2⋯H10*B*	2.42	− 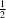 + *x*, 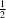 + *y*, *z*
H16⋯O24	2.73	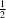 + *x*, 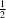 + *y*, *z*
H19*A*⋯N3	2.71	− 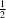 + *x*, 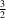 − *y*, − 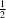 + *z*
S9⋯H10*A*	2.72	*x*, 1 − *y*, 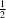 + *z*
S9⋯H21*B*	2.98	1 + *x*, 1 − *y*, 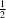 + *z*
H6⋯H16*A*	2.49	− 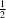 + *x*, 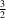 − *y*, 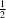 + *z*
N3⋯H15*A*	2.56	−1 + *x*, *y*, *z*
H8⋯O24	2.07	*x*, 1 − *y*, 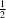 + *z*
H19*B*⋯H22*A*	2.58	*x*, *y*, *z*
H22*C*⋯H21*A*	2.31	*x*, 1 − *y*, − 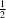 + *z*

**Table 3:** Experimental details

Crystal data	
Chemical formula	C_12_H_16_N_6_OS_2_·C_3_H_7_NO
*M*_r_	397.52
Crystal system, space group	Monoclinic, *C* *c*
Temperature (K)	100
*a*, *b*, *c* (Å)	8.4573 (1), 23.5853 (3), 11.0072 (1)
β (°)	111.749 (2)
*V* (Å^3^)	2039.29 (5)
*Z*	4
Radiation type	Cu *K*α
μ (mm^−1^)	2.57
Crystal size (mm)	0.32 × 0.12 × 0.02

Data collection	
Diffractometer	XtaLAB Synergy, Dualflex, HyPix
Absorption correction	Gaussian (*CrysAlis PRO*; Rigaku OD, 2023 ▸)
*T*_min_, *T*_max_	0.658, 1.000
No. of measured, independent and observed [*I* > 2σ(*I*)] reflections	11141, 2776, 2751
*R*_int_	0.030
(sin θ/λ)_max_ (Å^−1^)	0.634

Refinement	
*R*[*F* ^2^ > 2σ(*F* ^2^)], *wR*(*F* ^2^), *S*	0.032, 0.088, 1.07
No. of reflections	2776
No. of parameters	317
No. of restraints	377
H-atom treatment	H atoms treated by a mixture of independent and constrained refinement
Δρ_max_, Δρ_min_ (e Å^−3^)	0.30, −0.27
Absolute structure	Classical Flack method preferred over Parsons because s.u. lower
Absolute structure parameter	0.000 (16)

Computer programs: *CrysAlis PRO* (Rigaku OD, 2023 ▸), *SHELXT2019/1* (Sheldrick, 2015*a* ▸), *SHELXL2019/1* (Sheldrick, 2015*b* ▸), *ORTEP-3 for Windows* (Farrugia, 2012 ▸) and *PLATON* (Spek, 2020 ▸).

## supplementary crystallographic information

### Crystal data

**Table:**

C_12_H_16_N_6_OS_2_·C_3_H_7_NO	*F*(000) = 840
*M**_r_* = 397.52	*D*_x_ = 1.295 Mg m^−^^3^
Monoclinic, *C**c*	Cu *K*α radiation, λ = 1.54184 Å
*a* = 8.4573 (1) Å	Cell parameters from 9940 reflections
*b* = 23.5853 (3) Å	θ = 3.7–77.6°
*c* = 11.0072 (1) Å	µ = 2.57 mm^−^^1^
β = 111.749 (2)°	*T* = 100 K
*V* = 2039.29 (5) Å^3^	Plate, colourless
*Z* = 4	0.32 × 0.12 × 0.02 mm

### Data collection

**Table:**

XtaLAB Synergy, Dualflex, HyPix diffractometer	2776 independent reflections
Radiation source: micro-focus sealed X-ray tube, PhotonJet (Cu) X-ray Source	2751 reflections with *I* > 2σ(*I*)
Mirror monochromator	*R*_int_ = 0.030
ω scans	θ_max_ = 77.8°, θ_min_ = 3.8°
Absorption correction: gaussian (CrysAlisPro; Rigaku OD, 2023)	*h* = −10→10
*T*_min_ = 0.658, *T*_max_ = 1.000	*k* = −29→29
11141 measured reflections	*l* = −9→13

### Refinement

**Table:**

Refinement on *F*^2^	Hydrogen site location: mixed
Least-squares matrix: full	H atoms treated by a mixture of independent and constrained refinement
*R*[*F*^2^ > 2σ(*F*^2^)] = 0.032	*w* = 1/[σ^2^(*F*_o_^2^) + (0.0653*P*)^2^ + 0.7217*P*] where *P* = (*F*_o_^2^ + 2*F*_c_^2^)/3
*wR*(*F*^2^) = 0.088	(Δ/σ)_max_ < 0.001
*S* = 1.07	Δρ_max_ = 0.30 e Å^−^^3^
2776 reflections	Δρ_min_ = −0.27 e Å^−^^3^
317 parameters	Absolute structure: Classical Flack method preferred over Parsons because s.u. lower
377 restraints	Absolute structure parameter: 0.000 (16)

### Special details

**Table:**

Geometry. All esds (except the esd in the dihedral angle between two l.s. planes) are estimated using the full covariance matrix. The cell esds are taken into account individually in the estimation of esds in distances, angles and torsion angles; correlations between esds in cell parameters are only used when they are defined by crystal symmetry. An approximate (isotropic) treatment of cell esds is used for estimating esds involving l.s. planes.

### Fractional atomic coordinates and isotropic or equivalent isotropic displacement parameters (Å^2^)

**Table:**

	*x*	*y*	*z*	*U*_iso_*/*U*_eq_	Occ. (<1)
S2	0.28482 (9)	0.88049 (2)	0.35678 (8)	0.02715 (16)	
S9	0.62361 (9)	0.43859 (2)	0.67836 (8)	0.02788 (17)	
O18	0.3029 (2)	0.69867 (7)	0.22627 (17)	0.0208 (4)	
N1	0.3182 (3)	0.82853 (9)	0.5810 (2)	0.0250 (4)	
H1A	0.321 (5)	0.7946 (11)	0.623 (4)	0.035 (10)*	
H1B	0.277 (5)	0.8600 (14)	0.609 (4)	0.050 (12)*	
N3	0.3551 (3)	0.77463 (9)	0.4201 (2)	0.0193 (4)	
H3	0.338 (4)	0.7707 (16)	0.335 (2)	0.030 (9)*	
N4	0.3832 (3)	0.72789 (9)	0.4985 (2)	0.0194 (4)	
N7	0.5105 (3)	0.58678 (9)	0.5112 (2)	0.0206 (4)	
N8	0.5322 (3)	0.54302 (9)	0.5966 (2)	0.0237 (4)	
H8	0.494 (4)	0.5441 (15)	0.660 (3)	0.025 (8)*	
N10	0.6502 (3)	0.49254 (10)	0.4741 (3)	0.0304 (5)	
H10A	0.637 (4)	0.5197 (13)	0.412 (3)	0.028 (8)*	
H10B	0.689 (5)	0.4610 (13)	0.447 (4)	0.042 (10)*	
C2	0.3196 (3)	0.82596 (10)	0.4617 (3)	0.0196 (5)	
C5	0.4275 (3)	0.68202 (10)	0.4549 (2)	0.0178 (5)	
C6	0.4552 (3)	0.63348 (11)	0.5415 (3)	0.0211 (5)	
H6	0.432845	0.636140	0.619743	0.025*	
C9	0.6017 (3)	0.49391 (11)	0.5749 (3)	0.0245 (5)	
C11	0.4518 (3)	0.67600 (10)	0.3245 (2)	0.0180 (4)	
H11	0.462814	0.634948	0.306311	0.022*	
C12	0.6115 (14)	0.7087 (7)	0.3235 (18)	0.017 (2)	0.57 (4)
C13	0.7717 (16)	0.6857 (6)	0.3961 (17)	0.019 (2)	0.57 (4)
H13	0.781040	0.650478	0.440053	0.023*	0.57 (4)
C14	0.9181 (14)	0.7156 (8)	0.4026 (14)	0.025 (2)	0.57 (4)
H14	1.027777	0.700727	0.451350	0.030*	0.57 (4)
C15	0.9025 (17)	0.7670 (7)	0.3378 (13)	0.024 (2)	0.57 (4)
H15	1.001690	0.787616	0.343977	0.029*	0.57 (4)
C16	0.7452 (17)	0.7882 (6)	0.2650 (14)	0.0204 (19)	0.57 (4)
H16	0.736384	0.822536	0.217843	0.025*	0.57 (4)
C17	0.5977 (16)	0.7599 (7)	0.2590 (16)	0.0155 (19)	0.57 (4)
H17	0.488928	0.775672	0.211265	0.019*	0.57 (4)
C12A	0.618 (2)	0.7024 (9)	0.339 (2)	0.017 (3)	0.43 (4)
C13A	0.771 (2)	0.6743 (9)	0.411 (2)	0.022 (3)	0.43 (4)
H13A	0.769780	0.639161	0.452901	0.026*	0.43 (4)
C14A	0.9259 (19)	0.6998 (9)	0.4195 (19)	0.023 (2)	0.43 (4)
H14A	1.030313	0.681309	0.467394	0.027*	0.43 (4)
C15A	0.9282 (19)	0.7516 (9)	0.3588 (17)	0.020 (2)	0.43 (4)
H15A	1.033708	0.767984	0.365263	0.025*	0.43 (4)
C16A	0.780 (2)	0.7788 (8)	0.290 (2)	0.023 (3)	0.43 (4)
H16A	0.781356	0.814671	0.251521	0.028*	0.43 (4)
C17A	0.625 (2)	0.7534 (9)	0.277 (2)	0.021 (3)	0.43 (4)
H17A	0.521200	0.771477	0.225499	0.025*	0.43 (4)
C19	0.2808 (3)	0.67848 (12)	0.0984 (3)	0.0257 (5)	
H19A	0.191339	0.700524	0.032715	0.039*	
H19B	0.248038	0.638391	0.091007	0.039*	
H19C	0.387790	0.682681	0.084046	0.039*	
O24	0.2984 (3)	0.43619 (10)	0.2226 (3)	0.0449 (6)	
N20	0.1627 (4)	0.49731 (13)	0.3120 (3)	0.0405 (6)	
C21	0.0784 (5)	0.50587 (19)	0.4044 (5)	0.0551 (10)	
H21A	0.075716	0.469985	0.448488	0.083*	
H21B	−0.038264	0.519122	0.357418	0.083*	
H21C	0.140992	0.534251	0.469463	0.083*	
C22	0.1605 (5)	0.54375 (17)	0.2284 (4)	0.0503 (9)	
H22A	0.259076	0.541176	0.202153	0.075*	
H22B	0.165269	0.579467	0.275069	0.075*	
H22C	0.055571	0.542499	0.150377	0.075*	
C23	0.2273 (5)	0.44708 (17)	0.3000 (4)	0.0456 (8)	
H23	0.218115	0.417283	0.355074	0.055*	

### Atomic displacement parameters (Å^2^)

**Table:**

	*U*^11^	*U*^22^	*U*^33^	*U*^12^	*U*^13^	*U*^23^
S2	0.0435 (3)	0.0159 (3)	0.0262 (3)	0.0076 (2)	0.0178 (3)	0.0043 (2)
S9	0.0488 (3)	0.0145 (3)	0.0281 (3)	0.0061 (2)	0.0234 (3)	0.0048 (2)
O18	0.0219 (7)	0.0219 (8)	0.0180 (9)	0.0023 (7)	0.0067 (6)	0.0003 (7)
N1	0.0383 (11)	0.0165 (9)	0.0250 (11)	0.0039 (8)	0.0172 (9)	0.0016 (9)
N3	0.0264 (9)	0.0145 (9)	0.0198 (10)	0.0028 (7)	0.0117 (8)	0.0030 (8)
N4	0.0233 (8)	0.0140 (9)	0.0230 (10)	0.0021 (7)	0.0112 (8)	0.0016 (8)
N7	0.0298 (9)	0.0144 (9)	0.0200 (10)	0.0022 (8)	0.0120 (8)	0.0017 (8)
N8	0.0390 (11)	0.0148 (10)	0.0236 (11)	0.0044 (9)	0.0191 (9)	0.0029 (9)
N10	0.0538 (14)	0.0157 (10)	0.0324 (13)	0.0092 (10)	0.0287 (11)	0.0052 (9)
C2	0.0205 (9)	0.0158 (11)	0.0232 (12)	0.0015 (8)	0.0090 (9)	−0.0009 (9)
C5	0.0221 (10)	0.0135 (10)	0.0192 (11)	0.0007 (8)	0.0093 (9)	0.0007 (8)
C6	0.0307 (11)	0.0158 (11)	0.0208 (11)	0.0010 (9)	0.0141 (9)	0.0012 (10)
C9	0.0345 (12)	0.0169 (12)	0.0248 (12)	0.0016 (9)	0.0141 (10)	0.0011 (10)
C11	0.0199 (9)	0.0165 (10)	0.0186 (11)	0.0023 (8)	0.0081 (8)	0.0007 (9)
C12	0.014 (3)	0.023 (5)	0.013 (4)	−0.002 (3)	0.005 (2)	0.002 (3)
C13	0.028 (3)	0.015 (4)	0.017 (4)	0.001 (2)	0.011 (2)	0.007 (3)
C14	0.020 (2)	0.031 (5)	0.023 (4)	0.004 (3)	0.007 (2)	0.004 (4)
C15	0.025 (4)	0.025 (5)	0.024 (4)	0.000 (3)	0.011 (3)	0.003 (3)
C16	0.025 (4)	0.021 (4)	0.017 (4)	−0.002 (3)	0.009 (3)	0.002 (3)
C17	0.019 (3)	0.016 (4)	0.013 (4)	0.000 (3)	0.008 (3)	0.004 (2)
C12A	0.033 (5)	0.013 (4)	0.011 (5)	0.004 (3)	0.014 (3)	0.000 (3)
C13A	0.019 (3)	0.028 (6)	0.020 (5)	0.004 (3)	0.008 (3)	0.005 (4)
C14A	0.023 (3)	0.025 (6)	0.021 (5)	0.003 (4)	0.008 (3)	0.004 (4)
C15A	0.018 (3)	0.022 (6)	0.020 (5)	0.002 (4)	0.006 (3)	−0.004 (4)
C16A	0.030 (5)	0.021 (5)	0.018 (5)	−0.004 (4)	0.009 (4)	0.003 (4)
C17A	0.022 (4)	0.020 (5)	0.019 (6)	0.008 (4)	0.006 (4)	0.001 (4)
C19	0.0297 (11)	0.0270 (13)	0.0184 (12)	0.0000 (10)	0.0065 (10)	−0.0003 (10)
O24	0.0539 (13)	0.0453 (14)	0.0487 (15)	0.0031 (11)	0.0343 (12)	0.0024 (11)
N20	0.0429 (13)	0.0399 (15)	0.0428 (16)	−0.0030 (11)	0.0206 (12)	−0.0034 (13)
C21	0.056 (2)	0.060 (2)	0.060 (3)	−0.0086 (19)	0.0335 (19)	−0.013 (2)
C22	0.0547 (19)	0.0407 (18)	0.059 (2)	−0.0030 (16)	0.0248 (17)	0.0019 (18)
C23	0.0524 (18)	0.0432 (18)	0.050 (2)	−0.0031 (15)	0.0299 (17)	−0.0010 (16)

### Geometric parameters (Å, º)

**Table:**

S2—C2	1.680 (3)	C15—C16	1.368 (9)
S9—C9	1.697 (3)	C15—H15	0.9500
O18—C11	1.426 (3)	C16—C17	1.394 (9)
O18—C19	1.430 (3)	C16—H16	0.9500
N1—C2	1.319 (4)	C17—H17	0.9500
N1—H1A	0.92 (2)	C12A—C17A	1.390 (14)
N1—H1B	0.92 (2)	C12A—C13A	1.407 (13)
N3—N4	1.365 (3)	C13A—C14A	1.413 (13)
N3—C2	1.366 (3)	C13A—H13A	0.9500
N3—H3	0.90 (2)	C14A—C15A	1.398 (12)
N4—C5	1.294 (3)	C14A—H14A	0.9500
N7—C6	1.288 (3)	C15A—C16A	1.361 (12)
N7—N8	1.362 (3)	C15A—H15A	0.9500
N8—C9	1.360 (3)	C16A—C17A	1.401 (13)
N8—H8	0.88 (2)	C16A—H16A	0.9500
N10—C9	1.318 (4)	C17A—H17A	0.9500
N10—H10A	0.91 (2)	C19—H19A	0.9800
N10—H10B	0.90 (2)	C19—H19B	0.9800
C5—C6	1.452 (3)	C19—H19C	0.9800
C5—C11	1.530 (3)	O24—C23	1.238 (4)
C6—H6	0.9500	N20—C23	1.332 (5)
C11—C12A	1.492 (19)	N20—C22	1.426 (5)
C11—C12	1.558 (13)	N20—C21	1.456 (5)
C11—H11	1.0000	C21—H21A	0.9800
C12—C17	1.384 (10)	C21—H21B	0.9800
C12—C13	1.402 (10)	C21—H21C	0.9800
C13—C14	1.404 (10)	C22—H22A	0.9800
C13—H13	0.9500	C22—H22B	0.9800
C14—C15	1.387 (9)	C22—H22C	0.9800
C14—H14	0.9500	C23—H23	0.9500
			
C11—O18—C19	112.21 (18)	C15—C16—H16	119.6
C2—N1—H1A	117 (3)	C17—C16—H16	119.6
C2—N1—H1B	121 (3)	C12—C17—C16	119.3 (8)
H1A—N1—H1B	119 (4)	C12—C17—H17	120.3
N4—N3—C2	120.8 (2)	C16—C17—H17	120.3
N4—N3—H3	120 (2)	C17A—C12A—C13A	119.3 (13)
C2—N3—H3	118 (2)	C17A—C12A—C11	121.0 (13)
C5—N4—N3	116.6 (2)	C13A—C12A—C11	119.7 (13)
C6—N7—N8	116.0 (2)	C12A—C13A—C14A	118.2 (12)
C9—N8—N7	118.7 (2)	C12A—C13A—H13A	120.9
C9—N8—H8	119 (2)	C14A—C13A—H13A	120.9
N7—N8—H8	123 (2)	C15A—C14A—C13A	121.2 (10)
C9—N10—H10A	128 (2)	C15A—C14A—H14A	119.4
C9—N10—H10B	124 (3)	C13A—C14A—H14A	119.4
H10A—N10—H10B	107 (4)	C16A—C15A—C14A	120.2 (10)
N1—C2—N3	117.3 (2)	C16A—C15A—H15A	119.9
N1—C2—S2	125.8 (2)	C14A—C15A—H15A	119.9
N3—C2—S2	116.9 (2)	C15A—C16A—C17A	119.5 (11)
N4—C5—C6	114.5 (2)	C15A—C16A—H16A	120.3
N4—C5—C11	125.7 (2)	C17A—C16A—H16A	120.3
C6—C5—C11	119.8 (2)	C12A—C17A—C16A	121.7 (12)
N7—C6—C5	119.3 (2)	C12A—C17A—H17A	119.2
N7—C6—H6	120.3	C16A—C17A—H17A	119.2
C5—C6—H6	120.3	O18—C19—H19A	109.5
N10—C9—N8	117.3 (2)	O18—C19—H19B	109.5
N10—C9—S9	123.8 (2)	H19A—C19—H19B	109.5
N8—C9—S9	118.9 (2)	O18—C19—H19C	109.5
O18—C11—C12A	117.2 (9)	H19A—C19—H19C	109.5
O18—C11—C5	106.77 (19)	H19B—C19—H19C	109.5
C12A—C11—C5	108.1 (10)	C23—N20—C22	121.8 (3)
O18—C11—C12	109.5 (6)	C23—N20—C21	121.2 (3)
C5—C11—C12	112.3 (8)	C22—N20—C21	116.8 (3)
O18—C11—H11	109.4	N20—C21—H21A	109.5
C5—C11—H11	109.4	N20—C21—H21B	109.5
C12—C11—H11	109.4	H21A—C21—H21B	109.5
C17—C12—C13	120.6 (9)	N20—C21—H21C	109.5
C17—C12—C11	121.8 (9)	H21A—C21—H21C	109.5
C13—C12—C11	117.5 (9)	H21B—C21—H21C	109.5
C12—C13—C14	118.9 (8)	N20—C22—H22A	109.5
C12—C13—H13	120.6	N20—C22—H22B	109.5
C14—C13—H13	120.6	H22A—C22—H22B	109.5
C15—C14—C13	119.9 (8)	N20—C22—H22C	109.5
C15—C14—H14	120.1	H22A—C22—H22C	109.5
C13—C14—H14	120.1	H22B—C22—H22C	109.5
C16—C15—C14	120.5 (8)	O24—C23—N20	125.1 (4)
C16—C15—H15	119.8	O24—C23—H23	117.5
C14—C15—H15	119.8	N20—C23—H23	117.5
C15—C16—C17	120.8 (8)		
			
C2—N3—N4—C5	−175.4 (2)	C5—C11—C12—C13	73.3 (16)
C6—N7—N8—C9	−175.4 (2)	C17—C12—C13—C14	0 (3)
N4—N3—C2—N1	1.7 (3)	C11—C12—C13—C14	−176.8 (14)
N4—N3—C2—S2	−179.27 (16)	C12—C13—C14—C15	0 (2)
N3—N4—C5—C6	−179.78 (19)	C13—C14—C15—C16	−1.5 (19)
N3—N4—C5—C11	0.4 (3)	C14—C15—C16—C17	2.8 (19)
N8—N7—C6—C5	−179.5 (2)	C13—C12—C17—C16	1 (3)
N4—C5—C6—N7	−175.5 (2)	C11—C12—C17—C16	178.0 (14)
C11—C5—C6—N7	4.3 (3)	C15—C16—C17—C12	−3 (2)
N7—N8—C9—N10	2.6 (4)	O18—C11—C12A—C17A	12 (2)
N7—N8—C9—S9	−178.22 (17)	C5—C11—C12A—C17A	−109 (2)
C19—O18—C11—C12A	79.2 (11)	O18—C11—C12A—C13A	−165.2 (16)
C19—O18—C11—C5	−159.5 (2)	C5—C11—C12A—C13A	74 (2)
C19—O18—C11—C12	78.8 (8)	C17A—C12A—C13A—C14A	1 (3)
N4—C5—C11—O18	−50.3 (3)	C11—C12A—C13A—C14A	178.2 (18)
C6—C5—C11—O18	129.9 (2)	C12A—C13A—C14A—C15A	0 (3)
N4—C5—C11—C12A	76.6 (9)	C13A—C14A—C15A—C16A	0 (2)
C6—C5—C11—C12A	−103.2 (9)	C14A—C15A—C16A—C17A	−2 (2)
N4—C5—C11—C12	69.6 (6)	C13A—C12A—C17A—C16A	−3 (4)
C6—C5—C11—C12	−110.1 (6)	C11—C12A—C17A—C16A	179.8 (19)
O18—C11—C12—C17	14.8 (19)	C15A—C16A—C17A—C12A	4 (3)
C5—C11—C12—C17	−103.6 (16)	C22—N20—C23—O24	4.0 (6)
O18—C11—C12—C13	−168.3 (13)	C21—N20—C23—O24	178.9 (4)

### Hydrogen-bond geometry (Å, º)

**Table:**

*D*—H···*A*	*D*—H	H···*A*	*D*···*A*	*D*—H···*A*
N1—H1*A*···N4	0.92 (3)	2.27 (4)	2.670 (3)	106 (3)
N1—H1*B*···S9^i^	0.92 (4)	2.54 (4)	3.450 (2)	172 (4)
N3—H3···O18	0.90 (2)	2.04 (3)	2.694 (3)	129 (3)
N8—H8···O24^ii^	0.87 (3)	2.07 (4)	2.847 (4)	149 (3)
N10—H10*A*···S9^iii^	0.91 (3)	2.72 (3)	3.570 (3)	156 (3)
N10—H10*B*···S2^iv^	0.91 (4)	2.42 (4)	3.322 (3)	175 (4)
C6—H6···O24^ii^	0.95	2.54	3.224 (4)	129
C11—H11···N7	1.00	2.42	2.853 (3)	105
C11—H11···S9^iii^	1.00	2.87	3.706 (2)	141
C17—H17···O18	0.95	2.45	2.787 (16)	101
C21—H21*B*···S9^v^	0.98	2.98	3.951 (5)	171

Symmetry codes: (i) *x*−1/2, *y*+1/2, *z*; (ii) *x*, −*y*+1, *z*+1/2; (iii) *x*, −*y*+1, *z*−1/2; (iv) *x*+1/2, *y*−1/2, *z*; (v) *x*−1, −*y*+1, *z*−1/2.
